# ‘At least our pituitaries will see the world’: Pituitary gland export from communist Bulgaria

**DOI:** 10.1017/mdh.2025.10029

**Published:** 2025-10

**Authors:** Daniela Koleva

**Affiliations:** Institute of Ethnology and Folklore Studies with Ethnographic Museum, Bulgarian Academy of Sciences, Sofia, Bulgaria

**Keywords:** Pituitary, Growth hormone, Bulgaria, Biolaw, Bioethics, Bioeconomy

## Abstract

The article focuses on the export of cadaveric pituitary glands from communist Bulgaria in the 1980s, used for the production of human growth hormone. The case is explored in the broader context of practices and transnational networks for the supply of pituitaries. Special attention is paid to the changes resulting from the turn to the production of recombinant growth hormone in the mid-1980s, which put an end to the international ‘market’ of pituitary glands. In the last sections, different perspectives are explored to make sense of the case under scrutiny: those of bioethics and biolaw, on the one hand, and of bioeconomy in a globalising world, on the other.

This article focuses on a specific case of transnational linkage and exchange, of a type that has so far largely remained under the radar of medical history research, although it has occasionally attracted public attention. Assuming that the Iron Curtain was in many respects a ‘nylon curtain’, that is, permeable, I am trying to understand what used to ‘flow’ through it and in which direction. The case under study refers to a crucial moment in the development of biotechnology geared to the treatment of pituitary dwarfism – the short stature and slow growth in children due to the deficiency of growth hormone. I am interested in the practices and networks for the supply of pituitary glands extracted from human cadavers for the production of the hormone, and the changes resulting from the turn to recombinant growth hormone in the mid-1980s. This turn brought an abrupt end to the international ‘market’ in pituitary glands and hence to the questionable practice of their harvesting and export by some Soviet bloc countries, which started in the 1970s and flourished throughout the 1980s.

I shall first describe this practice in two of these countries: Hungary and Bulgaria. While the Hungarian case is only briefly sketched, I have studied the Bulgarian one in some depth, based on the archives of the Medical Academy in Sofia and of the National Television, as well as on several interviews with contemporaries: medical personnel from the institution exporting the pituitaries, and the journalists who in 1991 reported the practice in national media. Then, I shall zoom out to the broader international context of the production and distribution of growth hormone and the sharing of related knowledge. Finally, different perspectives towards the case at hand will be discussed: of bioethics and biolaw on the one hand, and of bioeconomy on the other. I believe that their juxtaposition leads to a deeper understanding not only of this particular practice, but of some specificities of the scientific internationalism of the late years of the communist regimes in Europe.

## Travelling pituitary glands

In 1987, a public scandal broke out in Hungary, following an appeal to the Supreme Court: a renowned professor at the University of Szeged, head of its Endocrinology Department, was accused of an unusual barter.^1^ He was found to have acquired consumables for his research from the Italian pharmaceutical company Serono^2^ in exchange for pituitary glands (hypophyses) extracted from cadavers during autopsies in Hungarian hospitals. This weird cooperation had lasted for about ten years, resulting in the export of several thousand pituitary glands in the luggage of passengers crossing the Hungarian border. In 1986, the customs officers found the precious goods, and the ensuing investigation resulted in charges against the professor. What the Hungarian court had to decide, however, was not whether this was a misappropriation of human cadaver tissues without the knowledge of the relatives or the opportunity of opting out, but whether this activity had led to private financial gains, causing loss of profits for the state. As it turned out, awkwardly, the state-owned Human Vaccine Substance Production Company had engaged in the same type of transactions with a few Western European companies, including Serono. Both the professor and the state company argued that their only aim was to provide resources for medical research and to improve the quality of healthcare in Hungary. The public, however, was less interested in what the pituitaries had paid for; the scandal brought attention to the fact that Hungarian citizens had been organ donors for years without knowing it. With this ‘turbulent historical retrospect’, Judit Sandor opens her article on patients’ rights in the context of organ and tissue donation.^3^ I shall contextualise the case from a different perspective, as an example of a ‘bioeconomy’ of sorts taking place across the Iron Curtain.

A couple of years later, a similar scandal burst out in Bulgaria: on 25 July 1991, the national television aired a report on the patho-anatomy department of a Sofia hospital, followed by an interview revealing the practice of the extraction of organs from the bodies of patients who died in hospitals.^4^ The viewers saw the trepanation tools with which the organs were extracted and the facilities for their preservation. The extractor explained the procedure and cited the prices of the organs: five levs for dura mater, ten levs for tibia, etc. A few days later, the weekly humour and satire magazine *Starshel* [Hornet] published on its front page a brief but caustic text stating: ‘We knew that our minds belonged to the state, but honestly, we didn’t fancy that our bodies belonged to it as well, and in such a literal sense’. Referring to the TV broadcast, the author pitched into the export of ‘Bulgarian meat’: ‘We may not be able to travel abroad, but at least our pituitaries will see the world’, he concluded sarcastically.^5^ The accompanying cartoon showed a cow, a pig, a man and a woman flying up to heaven with angels’ wings attached to their shoulders. The pig explained to bewildered God: ‘Their offal was sold in Maleev’s slaughterhouse’. The ‘slaughterhouse’ in the case of hypophyses was the Research Institute for Endocrinology, Gerontology and Geriatrics (RIEGG), part of the Medical Academy (MA), and Atanas Maleev was the MA president and brother-in-law of Todor Zhivkov, head of the communist party and the state till 10 November 1989.

Based on RIEGG archives and other archival material, I have tracked down the beginning and the development of this practice in the 1980s. My interviews with the TV reporter and the *Starshel* author, as well as with two former RIEGG researchers, one of whom was involved in the collection and preservation of the hypophyses, added not only factual details but also a sense of the professional ethos and the personal perspectives of participants and witnesses.

The idea to export pituitary glands was launched by Ema Bozadjieva (1920–96), professor of endocrinology, who got her PhD degree in the Soviet Union in 1968 and was promoted to the position of Director of RIEGG in 1976. As a participant in the communist resistance during the Second World War and a member of the Bulgarian communist party, awarded with the prestigious Georgi-Dimitrov order, Bozadjieva was very well-connected in both professional and party circles. Her former subordinates described her as an excellent physician, very knowledgeable, fully devoted to her work, and an outstanding manager: energetic, enterprising and influential, albeit quick-tempered and nagging. Her research focused on the pathologies of the hypothalamic-pituitary and adrenal systems, in particular pituitary tumours and endocrine hypertension.^6^ Research on these topics was developed at RIEGG already in the early 1970s, which was reflected in its structure: one of its departments was the Diencephalic-pituitary one.^7^ Bozadjieva’s guiding motivation was reported to have been the need for resources to set up RIEGG laboratories: ‘An institute without labs is a village hospital,’ she used to say, according to the interviewees. They singled out her greatest success as head of RIEGG, where she established ten new laboratories and staffed them with top specialists. One of them was the Laboratory for Peptide Hormones, which is of interest here.

In a report to the Chairman of the Committee for Science and Technical Progress dated 27 June 1980, Bozadjieva presented a research theme for which she sought funding: ‘Isolation of somatotropic and gonadotropic hormones from human pituitary glands from cadavers and development of a production technology and dosage forms for their administering’. She explained that the somatotropic/growth hormone (GH) was a complex polypeptide compound, which was not yet synthesised chemically, even though its composition was known.^8^ Therefore, the treatment of pituitary insufficiency in children (the so-called hypopituitary dwarfism) depended on the extraction of GH from the pituitary glands of human cadavers, the hormone being species-specific. Bozadjieva defended her proposal, pointing out that in the Soviet Union, GH was produced and children were treated as of 1959–60. The GDR also produced its own GH but ‘refused’ its export to Bulgaria. This was why by the time the report was written, no treatment had been provided for Bulgarian children suffering from pituitary dwarfism.^9^ There is no clue in the report as to the number of those children, and hence the social significance of the issue. A 1982 survey identified a total of 209 such children and adolescents registered in the paediatric wards of the regional hospitals and a frequency of one out of 19,000 children.^10^

RIEGG focused primarily on such widespread and hence socially significant diseases as diabetes, hypertension, goitre, and atherosclerosis. Having meagre chances to garner support based on the social importance of the problem, Bozadjieva adopted a different tactic. She referred to the situation in Western countries, where there was a chronic shortage of GH.^11^ Hence, the financial argument followed: Bozadjieva estimated that on average, 15–20 thousand autopsies were carried out in Bulgaria per year, which would account for 15,000 pituitaries. The GH extracted from them would be more than enough for Bulgarian needs, and a substantial share of it could be exported. The average price of a pituitary at the international market being USD 8, Bozadjieva estimated that the annual revenue from the export would reach 200–300 thousand currency levs per year.^12^ In addition to the extraction of pituitary glands and clinical research to be performed at RIEGG, the proposal envisaged laboratory research, including experiments on animals and, last but not least, market-oriented production.

Bozadjieva was either unaware or preferred not to mention that some children actually did receive treatment at the Endocrinology clinic of the Research Institute of Paediatrics as part of a research project led by Dr Lilia Peneva (1929–2020). It started in 1970 with eight children and developed to include a total of 43 children and adolescents by 1984. They were treated with cadaveric GH produced in Bucharest, Romania, and in Dresden, GDR (Sotropin H), later also with Serono’s Grorm, received in exchange for pituitaries extracted from cadavers in MA clinics.^13^ While these details were missing from the report, the small-scale informal exchange of pituitaries must have provided a powerful incentive for its author: one of my interviewees remembered Peneva’s name and those of two other doctors who collected pituitaries in acetone and handed them to a lady who periodically came from Italy (presumably from Serono). He did not know of Peneva’s research; rather, he believed that the pituitaries were simply sold, and even mentioned the price of USD 2–2.5 per unit – a practice closely resembling the one in Hungary. At about the same time, a representative of the Swedish pharmaceutical company Kabi^14^ contacted RIEGG management and proposed a higher price, especially if the pituitaries were frozen. So, the chance was there to expand the enterprise and channel the revenue.

Bozadjieva and her team did not miss their chance: the reports in the subsequent years focus mostly on the export of pituitaries and less on the research carried out at RIEGG. Only in 1987 did the RIEGG management report that GH was obtained and purified, and tested on animals. A radioimmunoassay was developed and prepared in commercial form. Hard currency^15^ was secured for depyrogenation filters, which would allow the serial production of GH.^16^ There is no evidence in the archive that the Paediatric Endocrinology clinic benefited from these achievements, and if they had any consequences for treating the Bulgarian children suffering from hypopituitary dwarfism.

The boundaries between laboratory science and international trade proved rather porous. As can be seen from Bozadjieva’s further reports, her superiors seemed to be more interested in the export than in the research. Her report to the Director of the Import-Export department of the Medical Academy (MAIMEX), dated 28 January 1981, reveals her commitment to developing a large-scale harvesting of pituitary glands: following the instructions given by Kabi, she inspected a few regional hospitals to organise the extraction and the preservation of pituitaries. Their extraction did not create any problems, as, like in Hungary, performing an autopsy was required in each case of a patient dying in a hospital.^17^ There was no legal obligation to seek consent from the relatives of the deceased, or even to inform them.^18^ Therefore, Bozadjieva focused on practicalities: she asked for a dedicated order of the MA President, Atanas Maleev, ‘to regulate the collection’ nationwide,^19^ and for thirty freezers to secure the harvesting of 10–15 thousand pituitary glands per year across the whole country.

The start of the collection campaign was promising: the partnership with Kabi worked smoothly, with their representative coming monthly to pick up the pituitaries and pay. RIEGG staff toured the country to collect them from the hospitals. At the next stage, two researchers visited Kabi laboratories in Sweden to get acquainted with GH production. In a letter to Maleev from 23 June 1982, Bozadjieva announced that 3000 pituitaries had already been collected for the first half of the year and urged him to order their harvesting all over the country. The price was already USD 16.5 per unit, but she reasoned that it would be economically more profitable not to export the unprocessed glands but rather a semi-finished product obtained from them.^20^ It was therefore necessary to equip a laboratory.^21^ This request must have been taken into account: the annual report for 1983 boasts that the plan for ‘hard currency’ was met, which was a chance for RIEGG to invest the revenue in the purchase of unique equipment. The collection of pituitaries was ‘rhythmic’ due to the young colleagues’ ‘responsible and conscientious’ participation.^22^ The laboratory was installed by Kabi. It was a simple and practical structure assembled of containers with four rooms equipped with air conditioning to maintain low temperatures. The quality of the product was thus guaranteed, and the revenue increased.

The exchange of pituitary glands for hard currency in the situation of growing national debt and chronic shortages was of great importance for MA. For 1985, the revenue from pituitaries was 702,000 currency levs, c. 30% of the total hard-currency revenue from MA’s production and international activities;^23^ for 1987, the share was 26%.^24^ The greater part of the earnings, as in the Hungarian case, was invested in equipment and consumables for research. Under the conditions of an over-centralised and inert planning system, this was not at all simple. In a series of reports to the President of MA, the RIEGG Director asked for permission to spend part of the foreign currency revenue for additional equipment and consumables, which had not been included in the plan but were urgently needed. By the early autumn of 1982, Bozadjieva reports, the export of pituitaries had yielded 76,249 currency levs; 45,313 were already allocated for the purchase of equipment and consumables, ‘the rest were profit’ that was placed at the disposal of MA management. Bozadjieva asked for permission to spend part of it on additional equipment. Since further hard currency income was expected till the end of the year, these expenses would not impair the profit, she promised.^25^

I could not find out if this request was approved or how long it took for the equipment to be bought and installed. As seen from other cases, the procedure was slow and cumbersome. First, the equipment had to be included in the plan for the next year (ideally – in the five-year plan), based on a proven need, i.e. that it was indispensable for the implementation of planned and approved research tasks; sometimes this also necessitated proof that equivalent equipment was not produced in any of the Soviet-bloc countries. Next, even if present in the plan, the purchase depended on the availability of funds, which were always in short supply, thus generating tough competition among heads of departments/institutes within the MA.^26^ The success often depended on the winners’ connections and positions in complex, largely informal, professional-political networks.^27^ In this situation, the capacity of RIEGG to garner foreign currency boosted its prestige among the other institutes and departments as a donor to the currency budget of the MA.

However, the situation worsened dramatically in the second half of the 1980s. In 1988, the new RIEGG Director Dragomir Koev fought for a budget of only 3000 currency levs for the Laboratory for Peptide Hormones in addition to the 7000 allocated to RIEGG as a whole.^28^ He explained that the sum was urgently needed for the import of materials and substances instrumental for the production of the radioimmunoassays planned for 1988. If the standard supply procedure were to be followed, these essential materials would arrive only in 1989, jeopardising the achievement of the plan. The reason was not that Koev lacked his predecessor’s authority (which he did, in fact); nor was it only the deepening economic crisis in the last years of the communist regime. A look at the reports shows the actual situation: while RIEGG increased the plan for the export of pituitaries to 850,000 currency levs (to honour the 13^th^ Congress of the Bulgarian Communist Party in 1986), only 44.89% of this amount was achieved.^29^ The negative tendency continued, down to a 10% execution of a plan already reduced to 400,000 currency levs in 1989.^30^ To explain this collapse, a broader international perspective is needed.

## Helping children to grow

The medicalisation of somatic realities, including short stature (especially for boys), started rather early in the twentieth century. As infant mortality declined, paediatricians’ focus shifted towards issues of ‘normal’ physical and psychological development.^31^ GH therapy attracted the attention of researchers and the media by the 1930s.^32^ Because of the hormone’s species-specificity, it had to be isolated from the pituitary glands of human cadavers and purified for therapeutic use. This only became possible in the 1950s. The preparations followed two methods, known as the Raben and Wilhelmi methods, whereby ‘[a]cetone-dried powdered pituitary tissue was subjected to repeated organic solvent extractions (Raben) or alkali and acid precipitations (Wilhelmi)’.^33^ After 1975, the latter method was modified using frozen glands and a more gentle extraction procedure.^34^ In the USA, where GH therapy was firmly established by the early 1960s, the supply of pituitaries depended on people’s decision to donate their organs post mortem, especially after the adoption of the Uniform Anatomical Gift Act (UAGA) in 1968.^35^ The National Pituitary Agency (a federal body established in 1963 to standardise the collection, production and distribution of human GH)^36^ and the Human Growth Foundation (an organisation of parents of small-stature children) energetically campaigned for the donation of pituitary glands. Still, the latter were in chronic short supply. Initially, the therapy required 1 mg of GH per day, which was extracted from one gland; so 365 pituitaries were needed per patient per year. About 10,000 of them were available in total.^37^ That is, the annual yield of pituitaries in Bulgaria, with its population of slightly over eight million, was comparable to or exceeding that of the USA.

In the 1970s, extraction and purification were ameliorated and ‘only’ 50 to 200 extracts were needed to provide one child with one year of treatment.^38^ Still, the therapy was hardly available to all those who wanted it. Doctors had to single out those children who were in the greatest ‘need’ for treatment, which led to a ‘rationing mentality’ and affected the very definition of the condition as against the ‘normal’ state.^39^ Patients were recruited as participants in research projects, as was the case at the Research Institute of Paediatrics in Sofia. Nevertheless, their treatment was often interrupted because of a GH shortage.^40^ Only in the mid-1970s, with the introduction of commercial pituitary GH, did the supply increase. Its main producers were Serono and Kabi. The treatment with these medications cost between USD 9,000 and USD 20,000 per year.^41^ The high cost, determined by the low supply, was yet another problem both public health agencies and patients’ families faced.

Since the supply of pituitary GH products was restricted due to the limited number of human hypophyses, both scientists and pharmaceutical companies turned to genetic engineering techniques in order to produce synthetic GH. In particular, Kabi, which dominated the global GH market, joined forces in 1978 with California-based laboratory Genentech (GENetic ENgineering TECHnology)^42^ to obtain genetically modified bacteria capable of producing human growth hormone. Kabi funded the venture and supplied human pituitary material. It might be that some of the hypophyses extracted in Bulgarian hospitals and sold to Kabi were allocated to this research. The hormone was synthesised in 1979 and submitted to the US Food and Drug Administration (FDA) for approval.^43^ The synthetic GH was expected to compete with the pituitary GH, which was considered more ‘natural’, and whose additional advantage was that it was often distributed by government agencies free of charge.

The technology was new, and the approval necessitated extensive trials over a relatively long period of time. However, the FDA trials ended abruptly, and the approval was urgently granted in October 1985, in the midst of a crisis triggered by an alarming discovery: a link was proven to exist between cadaver GH treatment and cases of a rare and fatal brain disease, Creutzfeldt-Jakob disease (CJD). The victims were young adults from several countries, including the USA, the UK, and France. The problem was very serious, bearing in mind that for nearly thirty years, more than 27,000 children worldwide had already been treated with cadaveric GH.^44^ The public outcry was significant, due to both the young age of the victims and the long incubation period, which led to the perception of the disease as a constant hidden threat for the patients and a potential risk for their families and the wider public.^45^ Furthermore, the iatrogenic nature of the CJD made it look like ‘friendly fire’, which undermined public trust in biomedicine.^46^ Under the pressure of the controlling agencies, the pharmaceutical companies had to withdraw their cadaver-based products from the market. However, with thousands of children’s treatment interrupted, the FDA and the controlling bodies in other countries were themselves under pressure to provide an alternative. As a result, the synthetic (recombinant) GH (rGH) was speedily approved, and the expected bias against its ‘artificiality’ did not materialise. By the late 1980s, the treatment with cadaveric GH was discontinued, first in the USA and the UK in 1985, then in the rest of the world as well. Its replacement, the rGH, is considered to have ‘an enviable track record of safety’.^47^ Still, lessons learned from the CJD crisis led to acute awareness of potential adverse effects. Therefore, many countries established registries of patients treated with rGH to monitor potential side effects.^48^

These developments explain the problems RIEGG was facing in meeting its export plans in the second half of the 1980s. Even after the planned revenue from the export of pituitaries was decreased, the failure to meet it was spectacular, due to the rapidly shrinking demand. This meant less foreign currency, less laboratory consumables and reagents and consequently, failure to perform planned research tasks. The plan was geared to the major political event in communist Bulgaria, the 13^th^ congress of the Bulgarian communist party, and did not even take notice of, let alone analyse, the actual situation of the therapy based on pituitary GH. In 1991, in the wake of the public scandal, the RIEGG Director informed his superiors that legal advice had been sought and a change of the law was needed in order to continue the export of pituitaries. However, there was no more demand for them.

## Legal and ethical dilemmas of cadaveric donation

The hypophyses case can be viewed from different perspectives. The broader story is one of a biotechnological breakthrough: a better and safer product was invented, allowing many more patients to receive effective treatment.^49^ As of 1993, Bulgaria started to import rGH, and Bulgarian children suffering from hypopituitary dwarfism received treatment with good results. In the subsequent years, patients with other conditions related to GH deficiency became eligible for such treatment.^50^ The availability of appropriate drugs played back onto the definition of the norm and pathology in relation to the levels of hormone secretion and hence the criteria for eligibility for treatment.^51^ The cut-off GH level separating normality from GH deficiency fell from 10 to 7μg/L.^52^ The question was now where to draw the line between the norm and pathology, therapy and cosmetic enhancement. The kind of knowledge needed to implement such differentiations went beyond laboratories into the legal and moral sphere.

The hypophyses case, however, poses another type of ethical issue. It can be viewed in the context of decades-long legal and ethical discussions on the utilitarian treatment of the donors. In the situation of short and unpredictable supply of organs and tissues for transplantation, some legal scholars have argued for ‘organ conscription’, i.e. a universal and mandatory post-mortem draft of organs and tissues, seeing the law as a mediator between the medical needs of the living and deep-rooted sentiments towards the dead, and insisting on its role to ‘implement common will and wisdom where voluntary incentive is lacking’.^53^ Such a position views the human body as a kind of natural resource ‘on extended loan from the biomass’ to which it returns after death.^54^ Its proponents argue for the ‘automatic’ availability of cadaver organs,^55^ prioritising (not without good reason) the needs of the living over the interests of the dead. Others, however, have emphasised the integrity of the human body. They have pointed to the specific ontological status of the corpse, asking if the human body turns into an object after death, and, if so, who has ownership rights on that object.^56^ Still others have argued that a corpse ‘can be said to have limited human rights, “passive” rights, to be sure, but rights nonetheless’ since, before the burial ritual, which delivers it to the world of the dead, ‘the corpse remains to some degree a member of the living human community’.^57^ Its ‘rights to bodily integrity, dignity, and respect’ are ‘a reinscription of obligations and expectations that constitute social life’.^58^ Therefore, the dead body does have specific rights and ‘an ethical status above that of property’.^59^ In any case, as many of these scholars agree, ‘economic considerations cannot overrule ethical ones’.^60^

Coming back to the hypophyses case in Hungary and Bulgaria, a conclusion that the totalitarian communist states disposed of the bodies of their citizens as if they were yet another state property would be a hasty and perhaps a superficial one. Indeed, the practice of organ and tissue harvesting for purchase by pharmaceutical companies, based on the assumption of tacit consent, was not limited to communist and so-called ‘Third World’ countries. After the scandals in Hungary and Bulgaria, another one burst out in Ireland in 1998, when it became known that in the 1970s, pharmaceutical companies had paid pathologists to extract pituitary glands and other organs from cadavers, especially those of children, without informing their parents. The price they were paid for a pituitary gland in 1978 was £1.50, and in 1985 it grew to £3.50. The families of the deceased children established the ‘Parents for Justice’ group to investigate the issue, which they defined as ‘the worst kind of theft’. Although at the time there had been no explicit regulation requiring informed consent, the practice was criticised on ethical grounds as a violation of the public’s trust in biomedicine and condemned as ‘dark age medicine’.^61^

The debate on public trust in medicine and its institutions and procedures was also simmering in the USA, although there, the supply of pituitaries was considered to have been better regulated. Not so long ago, the former director of the National Pituitary Agency (NPA) admitted that the extraction and collection of pituitaries had been ‘a tale of intrigue and secrecy’.^62^ The extractors were paid $2 for the collection, storage and shipment of a pituitary gland to the NPA. However, the competition for pituitaries was so strong that a black market developed. As it turned out, NPA was not always successful in its regulating and standardising role, which led to ‘questionable occurrences in conjunction with the NPA’s collection’.^63^

The medicalisation of death and its sequestration from everyday life gives real, albeit sometimes unacknowledged, power to medical professionals and institutions over dead bodies. To control this power, so-called anatomy laws were adopted in most countries, regulating the acquisition of organs and tissues. While the Universal Anatomy Gift Act (1968) and the National Organ Transplantation Act (1984) in the USA encouraged voluntary donation, many European countries (Austria, Belgium, Finland, France, Greece, Yugoslavia and others) adopted the principle of ‘presumed consent’ whereby organs could be harvested unless the deceased had, in their lifetime, explicitly refused to donate their organs.^64^ The principle of presumed consent and its alternatives, ranging from altruistic donation to routine retrieval, have generated a lot of discussion in Western Europe and the USA on how to balance between the need to eliminate the shortage of organs and the need to preserve the autonomy of the individual.^65^

These discussions did not resonate on the Eastern side of the Iron Curtain until well after the end of the communist regimes. What pathologists at Bulgarian hospitals did in the 1980s, extracting pituitary glands, was explicitly allowed by Article 34 of the Public Health Act then in force, which stipulated that organs and tissues for transplantation could be extracted from cadavers in hospitals. It also stated explicitly that the consent of the next of kin was not required.^66^ The legal regulation thus precluded an ethical perspective: harvesting cadaver organs and tissues was part of the medics’ job, it was reduced to a technological issue regulated by a protocol defining the criteria for identifying brain death, who was authorised to establish it and what the ensuing procedures were.^67^ Lilia Peneva openly stated that most of the GH she used was acquired in exchange for human pituitaries.^68^ Ema Bozadjieva and her team had to worry mainly about the organisation of the collection and the preservation of the pituitaries. And that was what they did, diligently and rather successfully. However, what the law and the ensuing regulation actually allowed was to extract organs and tissues for the sole purpose of transplantation. Only in 1991 was harvesting cadaver organs for ‘other therapeutic purposes, as well as scientific and educational purposes of health care’ allowed.^69^ As for the shipment of the pituitaries abroad, this matter fell out of legal regulations: it was not expressly forbidden, but not allowed either. Even less did the law discuss the question of potential profits from organ exchange and who was entitled to them.

Something is troubling about the trade in human tissues and organs, even if extracted from a cadaver. This is how the journalists – the TV reporter and the *Starshel* author – saw and presented the hypophyses case in 1991: as ethically, rather than legally, indefensible.^70^ The trouble seems to have deep roots in culture, in particular in beliefs that the dead body is entitled to some respect, and that extracting tissues and organs is a form of exploitation, which erodes social values; that it violates bodily integrity and eventually undermines trust in medicine and science in general. In the words of John Moore, a patient whose blood products were patented in the USA, ‘[t]hey view me as a mine from which to extract biological material’.^71^

Whether or not the extraction of organs and tissues is indeed exploitation and/or expropriation will depend on how the human body is seen. If a dead body were defined as refuse, which is freely available to be ‘recycled’, then its use for research and even commercial purposes would not be problematic. But if the organ/tissue is considered to have an inherent value as part of the human being, then its use, if done without the individual’s consent, would be ambiguous, to say the least. The intricacy of the hypophyses case comes from its shaky ground: the distinction between ‘that which is not human–ownable, tradeable, commodifiable – and that which is human – not legitimate material for such commodification – no longer seems stable’.^72^ In the case of harvesting cadaver organs, the families of the deceased were deprived of agency. In communist Bulgaria (as in Hungary), there were no civic organisations to raise the issue, no spokespersons to represent the interests of potential donors, and no civil society to initiate a discussion. In Hungary, the problem became known to the public only when it reached the Supreme Court; in Bulgaria, even later, after the start of the democratic changes. Furthermore, the export of the organs, performed by a public institution, fell into a legal ‘grey zone’. As seen from the Hungarian case, court proceedings were undertaken against the professor, i.e. the individual, but not against the institution engaged in the same kind of morally questionable and legally problematic activity. That is, state institutions could, and as a matter of course did, engage in practices for which citizens were brought to court. Viewed from this perspective, the pituitary case reveals the hypocrisy of the regime and the impossibility of civic action.

## Bioeconomy in a globalising world

Another perspective towards this case, one that I think is closer to the mentality and the situation of the professionals who were engaged in the collection of the pituitaries, foregrounds the asymmetric economic relations between the capitalist and the communist countries.^73^ The efforts to advance medical research, both in Bulgaria and in Hungary, were hindered by chronic shortages of resources. While Genentech and other laboratories had their research directly, and often generously, funded by public and private bodies, in particular by pharmaceutical companies,^74^ RIEGG researchers and their Hungarian colleagues obtained their incomparably more modest resources through a slow and clumsy system of state funding, which was, in most cases, inadequate. The lack of resources, the disarray resulting from the centralised planning and the complicated decision-making procedures defined the specificity of their daily situation at work. Therefore, the exchange of biological ‘raw material’ for additional funding was instrumental if research was to go on.^75^ Such exchanges proved feasible at the international markets, and both partners, Kabi and RIEGG, seem to have benefited from them. Unlike the journalists, my interviewees who used to work at RIEGG did not seem to have faced ethical conundrums – neither before, nor now; instead, they remembered practical problems that they had to solve, such as the production of ‘dry ice’ for preserving the pituitaries, and their own and their colleagues’ ingenuity in coping with obstacles. In retrospect, they seemed pleased and proud to enumerate the items of equipment they had managed to acquire with ‘pituitary money’ and the good uses they had made of it. Indeed, Bulgarian endocrinologists did conduct their own research in endocrine physiology.^76^ They apparently managed to acquire a better understanding of GH physiology, the hormones of the pituitary, etc. In 1986, in honour of the 13^th^ congress of the communist party, RIEGG director proudly reported that the Laboratory for Peptide Hormones had developed a technology for the isolation of pituitary hormones for diagnostic purposes and that its testing was underway. Such a technology was obviously not a worldwide novelty, but RIEGG researchers had limited opportunities to benefit from international research collaborations, except those with their colleagues from the Soviet Union and other Soviet-bloc countries. Only in 1991 was it possible to send two researchers to the USA to deepen their knowledge of peptide hormones.^77^

Looking at the broader context, the potential for scientific advancement was limited by factors beyond scarce resources and economic asymmetry. Something game-changing was happening beyond RIEGG walls, beyond Bulgaria, on the other side of the Iron Curtain: bioscience was undergoing ‘changes and mutations in the institutional structure and the functioning’,^78^ leading to a relocation of the governance of scientific research, reorientation of science policies and priorities that changed the landscape of biotechnological research. As a result, bioscience was no longer a mere academic science but rather a hybrid ‘post-academic’ one, where research became part of a larger cycle, supported financially and administratively by bodies whose motives and interests went beyond the production of knowledge: ‘Postacademic science becomes more entangled with “trans-epistemic” issues, involving societal, environmental and humanistic values. […] The world where research is to be applied is already highly structured. That is, the problems to be tackled will normally be set and funded by their organisational “owners”, such as industrial firms, government departments, health services, etc.’^79^ Post-academic science, broadly speaking, is related to social interests – in this case, helping children grow to ‘normal’ heights. (The social construction of this ‘normality’, and the whole ‘ethics of normalcy’ (Rose) is a different question.^80^) Thereby, it is also related to state institutions, businesses and markets.^81^

The idea of post-academic science leads to yet another possible frame to make sense of the pituitary case, namely the concept of biocapitalism as used in the past few years to ‘map the growing significance of the life sciences and biotechnology as an innovation within late capitalism that controls, changes and experiments with the material basis of life’.^82^ That is, a material (organ, tissue), which is not directly available but is derived from the human body through special technological procedures, becomes a form of capital – ‘biocapital’, which is valorised and used for knowledge production, and subsequently perhaps applied for therapeutic purposes or incorporated into the pharmaceutical industry. Thus, ‘value in the market sense and value in the ethical sense co-constitute one another in biocapital’.^83^ In Rose’s felicitous phrasing, the ‘biological existence of human beings has become political in novel ways’.^84^ Building on Foucault’s thesis that Western societies live in a ‘biopolitical’ age, Rose conceptualises contemporary biopolitics, among others, as ‘molecular politics’ where not only the norm of individual health replaced that of the health of the population, but the ‘clinical gaze’ gave way to ‘molecularisation’ of how life was imagined: as sub-cellular processes controlled by a genome. This conceptualisation seems revealing to think of the development of GH production. The molecularisation of biology was a change not only in the epistemology but also in the technology of research: it could be moved from the laboratory into the pharmaceutical plant in the sense of not just producing for ‘the market’ but of shaping ‘the very direction, organisation, problem space and solution effects of the biology itself’.^85^ Thereby, biopolitics has transformed itself into bioeconomics.

On this side of the Iron Curtain, however, biosciences followed a different scenario. The academic (rather than post-academic) science practised at RIEGG was supposed to relate to its subject in a more ‘disinterested’ way, which had to be guaranteed by the central planning of both research priorities and the infrastructures needed for their implementation. As the pituitary case has shown, this was often not the case. The scarcity of resources and the arbitrariness of management pushed Bulgarian research institutions into the global bioeconomy. In spite of their asymmetric position on the global scale, this brought them both revenue and prestige at home. Thus, a practice was grafted, which was not in line with the announced ideological and political principles, in particular with the claim to socialism’s moral superiority, but proved both viable and vital in economic terms. The post-mortem extraction of human organs and tissues was legally justified, which made moral questioning irrelevant. The practitioners involved in this activity did not think of it in moral terms and did not face moral dilemmas. For them, the influx of foreign currency was both a corporate success (revenue plus prestige) and a personal academic and career accomplishment.

## Concluding remarks

This study does have its limitations: It delves into a specific case without establishing how typical or atypical it was; its institutional, political and economic context cannot be presented in all its complexity. In terms of method, the comparison between the country cases is limited to a few parallels or differences. Still, it offers some insights into the history of medicine during the Cold War: not only the ‘nylon’ qualities of the Iron Curtain, but also some specificities of the research organisation on both its sides, which often remain invisible due to their mundane nature.

Taken in its minute detail, the pituitary case exemplifies the difficulties of organising and developing scientific research under the conditions of centralised planning with a direct, often heavy-handed political and administrative intervention. Research organisations had to comply with the government-set priorities for which funding was allocated. Their abilities to launch projects of their choice, as the one on somatotropic hormones discussed here, were limited and depended on complex negotiations, often themselves dependent on good professional and political connections. Even if the project was approved, this did not mean that resources were secured for its implementation – this was yet another struggle. Paradoxically, the ‘plan’ sometimes brought chaos. The shortage of resources (‘hard currency’ in particular) led to a shift of priorities: instead of a supplement to laboratory research, as initially envisaged, the export of pituitaries grew into a project in its own right. Junior RIEGG staff had to invest their talent into solving the technical and logistical tasks of the collection and preservation of this valuable commodity. Last but not least, successful organisations were not entitled to decide on the use of the revenue they generated. They needed a central approval followed by an unwieldy administrative procedure.

Taken beyond the specific details, the pituitary case exhibits a dilemma, which has actually been at the core of the ethical issues outlined above: the clash between the advancement of medical research and therapy, on the one hand, and the entrepreneurial approach to the human body, on the other. An organ acquired commercial value, even if in a medical research context. RIEGG and its Hungarian counterpart, the Human Vaccine Substance Production Company, were positioned as suppliers of a valuable ‘raw material’ for research and production, rather than as partners in research or production. With the pharmaceutical industry increasingly funding medical research, researchers found themselves increasingly tied to commercial goals.^86^ Thereby, the zone of competition was entered rather than that of scientific collaboration. Indeed, the Iron Curtain proved to be porous, but in different ways: knowledge, technologies, products, and equipment passed from the West to the East, while raw and auxiliary materials travelled in the opposite direction. The development of rGH was an example of technology transfer, i.e. the implementation of a scientific discovery into industry to manufacture a commercial product. This was a different situation than the polio epidemics in the mid-twentieth century where a ‘common enemy’ was identified to fight against with joint forces.^87^ Indeed, both cases focused on children, the future of society, who had to be cared for. But the scale was dramatically different, and the problems of the children suffering from pituitary insufficiency became visible only when a scandal burst out, revealing mistakes or misuses. In the pituitary case, the situation was not so much one of a collegial collaboration but rather an asymmetric partnership, defined by economic competition no less than the quest for knowledge. Unlike fundamental research, or ‘academic’ science, the stakes in ‘post-academic’ science are not only scientific but also economic, depending on streamlined technology and leading to commercial success. Indeed, rGH did turn out to be ‘a very viable commercial product’ and Genentech’s ‘glorifying success’.^88^

Medical research, like all basic science focused on non-military applications, is in principle international. However, the post-Second World War scientific internationalism cannot be overestimated, even in an apparently ideologically neutral, humanist and universal field such as medicine, where circulation of knowledge and technologies, rather than competition, was perceived to be the norm. Ideology could easily be circumvented, but, as far as the convergence of basic science and commercial applications was concerned, this was obviously not the case. Behind the rivalry and the competition in ideology and politics on the global arena, and in parallel with the shared epistemic paradigms of academic science, certain branches of Soviet-bloc medical research found themselves involved in a global biocapitalist economy.

## Footnotes

1 I am grateful to Prof. Judit Sandor for sharing the details of this case.

2 Founded in 1906 in Rome; later relocated to Switzerland.

3

Judit Sandor , ‘Right to health and the human organ: Right as a consequence?’, Pravni Zapisi, 5, 2 (2014), 384–412 .

4
*Otzvuk* [Echo] commentary studio, BNT archive, 25 July 1991.

5 Rumen Belchev, ‘Spi spokoino, hipofizo moya!’, *Starshel*, 2373, 2 August 1991, 1.

6 Ema Bozadjieva, *Endokrinni hipertonii* (Sofia: Medicina i fizkultura, 1979); *idem*, Georgi Papazov , Hormoni i mineralen obmen (Sofia: Medicina i fizkultura, 1984); *idem*, Lyubomir Karagyozov , Tumori na hipofizata (Sofia: Medicina i fizkultura, 1987).

7 Report on the fulfilment of the plan for research of the Institute for Endocrinology, Gerontology and Geriatrics for 1972. Sofia State Archive, holding 2265 (RIEGG), inventory 3, archival unit 10, sheet 6.

8 The biochemical structure of the growth hormone was first identified in the early 1970s by C. H. Li and J. S. Dixon: C. H. Li and J. S. Dixon . ‘Human pituitary growth hormone. 32. The primary structure of the hormone’, Archives of Biochemistry and Biophysics, 146, 1 (1971), 233–65144027 10.1016/s0003-9861(71)80060-7.

9 Report by Prof. E. Bozadjieva, director of RIEGG, to the Chairman of the Committee for Science and Technical Progress, comrade Nacho Papazov ex. No. 694 / 27 June 1980, Sofia State Archive, holding 2265 (RIEGG), inventory 3, archival unit 31, sheet 14.

10 Lilia Peneva, *Hiposomatotropizam v detskata i yunosheskata vyzrast.* Unpublished Dr. Sci. dissertation, Sofia: MA, Research Institute of Paediatry, 1984, 88–9.

11 RIEGG management often used comparisons with other countries to push for more funding, a practice that was obviously not exceptional (cf. Paula A. Michaels , ‘Soviet medical internationalism amid destalinization, 1953-1958’, The Soviet and Post-Soviet Review, 50 (2023), 40–63, 58).

12 Sofia State Archive, h. 2265 (RIEGG), inv. 3, a.u. 31, sheet 15. The ‘currency lev’ was an accounting currency unit used for the recalculation of revenues and expenditures from foreign currencies into Bulgarian levs and vice versa. Its relation to Western currencies was arbitrarily defined each year and kept unrealistically low, and was highly differentiated by type of industry and type of currency. In the 1980s, the US dollar equivalent was close to double, i.e. 1 USD = 1.5–2 currency levs. I am indebted to Prof. Krassen Stanchev for these clarifications.

13 Peneva, *op. cit.* (note 10), 83, 193; *idem*, R. Slavova, Zh. Zhelev, ‘Lechenie s choveshki rastezhen hormon na detsa s hipopituitarizym’, *Pediatria*, 15, 2 (1976), 98–106, 99; *idem*, Zaboliavania na hipofizata III. Lechenie na hipofizarnia nanizym, *Pediatria*, 21, 6 (1982), 547–53.

14 Founded in 1931 in Malmö. Nowadays part of Pfizer.

15 ‘Hard currency’ was the term used to refer to Western currencies, typically US dollars and FRG marks. It was essential for the import of preferred, sometimes unique, equipment and consumables.

16 Information on the fulfilment of the Biotechnology Programme of MA by RIEGG in 1987. Holding 2265 (RIEGG), inv. 3, a.u. 33, sheet 69.

17 Public Health Act, *Darzhaven Vestnik* [State Gazette] No 88, 1973, article 37. According to the extractor interviewed in *Otzvuk* studio, the ‘big shots’, i.e. high-ranking party and state officials, were exempt from autopsy.

18
*Ibid.*, article 34(2).

19 Sofia State Archive, holding 2265 (RIEGG), inv. 3, a.u. 13, sheet 2.

20
*Ibid.*, a.u. 7, sheet 40–41.

21
*Ibid.*, a.u. 50, sheet 1.

22
*Ibid.*, a.u. 26, sheet 8, 10.

23 ‘Report on the fulfillment of the currency plan of MA for 1985’, Sofia State Archive, holding 3863 (MAIMEX), inv. 4, a.u. 16, sheet 21.

24 ‘Report on the revenues in non-socialist currency on MA’s bank account in 1987’, *ibid.*, sheet 46.

25 Sofia State Archive, h. 2265 (RIEGG), inv. 3, a.u. 7, sheet 48.

26 Repeated complaints can be found in the reports and the minutes of RIEGG assemblies that the system of planning and supply of laboratory consumables was ‘rather inert and does not meet our needs’ (Sofia State Archive, h. 2265 (RIEGG), inv. 1, a.u. 9, sheet 28a) and that ‘we plan research tasks for the next year necessitating relevant equipment, which is also included in the plan, but we receive it in 3-4 years’ (Sofia State Archive, h. 2265 (RIEGG), inv. 3, a.u. 4, sheet 7).

27 On the importance of personal connections, see Daniela Koleva, Ignat Petrov, ‘Socialist gerontology? Or gerontology during socialism? The Bulgarian case’, *History of the Human Sciences*, 36, 3–4 (2023), 178–201, https://doi.org/10.1177/09526951231178434.

28 Sofia State Archive, h. 2265 (RIEGG), inv. 3, a.u. 7, sheet 4-5.

29 Sofia State Archive, h. 3863 (MAIMEX), inv. 4, a.u. 16, sheet 34.

30
*Ibid.*, sheet 50.

31 On the medicalisation of social stigmas in relation to the development of the GH industry, see Aimee Lynn Medeiros, *Heightened Expectations: The History of the Human Growth Hormone Industry in America.* UCSF Electronic Theses and Dissertations (2012), https://escholarship.org/uc/item/5n8021rj.

32 Dr Harvey Cushing coined the term in 1909, and the growth-promoting effects of GH were proven in a groundbreaking study by H. M. Evans and his team in 1921 ( Martin O. Savage , Abdullah Alherbish , ‘Growth hormone therapy for paediatric growth disorders: The past, present and future’, Dr. Sulaiman Al Habib Medical Journal, 2, 1 (2020), 4–9, 10.2991/dsahmj.k.200227.001 .

33 C.J.Ellis, H. Katifi, R.O. Weller, ‘A further British case of growth hormone induced Creutzfeldt-Jakob disease’, *Journal of Neurology, Neurosurgery, and Psychiatry*, 55 (1992), 1200–2, 1201.

34
*Ibid.*, 1201. For details see R. Lumley Jones , G. Benker , P.P. Salacinski , T.J. Lloyd and P.J. Lowry , ‘Large-scale preparation of highly purified pyrogen-free human growth hormone for clinical use’, Journal of Endocrinology, 82 (1979), 77–86 479737 10.1677/joe.0.0820077.

35
http://www.uniformlaws.org/shared/docs/anatomical_gift/uaga%201968_scan.pdf, accessed 28 April 2025.

36 Renamed as National Hormone and Pituitary Program, NHPP. It repeatedly issued appeals to pathologists to engage in the collection of pituitaries, and to citizens to donate posthumously their pituitaries in order to help dwarfed children. Similar government-sponsored agencies were established in other countries, such as the UK, Canada, France, Australia, and Israel.

37

Robert M. Blizzard , ‘History of growth hormone therapy’, Indian Journal of Pediatrics, 79, 1 (2012), 87–91, 88, 10.1007/s12098-011-0609-4 22081428 .

38 Medeiros, *op. cit.* (note 31), 151.

39

Maureen D. McKelvey , Evolutionary Innovations: The Business of Biotechnology. (Oxford: Oxford University Press, 2000), 231 .

40

Evan Graber , Edward O. Reiter and Alan D. Rogol , ‘Human growth and growth hormone: From Antiquity to the recombinant age to the future’, Frontiers in Endocrinology, 12 (2021), 1–19, 6, 10.3389/fendo.2021.709936 PMC828742234290673. This was also the case in Sofia, as Peneva repeatedly explained. The irregular supply and the high price of the GH led her to experiment with intermittent treatment after the first year. Peneva, *op. cit.* (note 10), 337; *op. cit.* (note 13), 551; Peneva et al., *op. cit.* (note 13), 99.

41 Medeiros, *op. cit.* (note 31), 184.

42 Genentech, in collaboration with Eli Lilly, developed recombinant human insulin, the first biotech therapy approved by the FDA in 1982 ( Priya Venkasten , Michael A. Peters , ‘Biocapitalism and the politics of life’, Geopolitics, History, and International Relations, 2 (2010), 100–22, 107).

43 Medeiros, *op. cit.* (note 31), 186–8.

44 Until 2021, more than 250 individuals who had received cadaveric GH treatment were diagnosed with CJD worldwide (Graber et al., *op. cit.* (note 40), 6). Some studies established a possible genetic predisposition to iatrogenic CJD ( John Collinge , Mark S. Palmer , Aidan J. Dryden , ‘Genetic predisposition to iatrogenic Creutzfeldt-Jakob disease’, The Lancet, 337, (1991), 1441–210.1016/0140-6736(91)93128-v1675319), others pointed out that most CJD patients received treatment before 1977, the year when the method of extraction was significantly improved ( Joseph Y. Abrams , Lawrence L. Schonberger , Ermias D. Belay , Ryan A. Maddox , Ellen W. Leschek , James L. Mills , Diane K. Wysowski , Judith E. Fradkin , ‘Lower risk of Creutzfeldt-Jakob disease in pituitary growth hormone recipients initiating treatment after 1977’, Journal of Clinical Endocrinology & Metabolism, 96, 10 (2011), 1666–910.1210/jc.2011-135721816775). Still, a much-discussed French study revealed that the new method did not fully exclude contamination ( T. Billette de Villemeur , J.P. Deslys , A. Pradel , C. Soubrié , A. Alpérovitch , M. Tardieu , J.L. Chaussain , J.J. Hauw , D. Dormont , M. Ruberg , Y. Agid , ‘Creutzfeldt-Jakob disease from contaminated growth hormone extracts in France’, Neurology, 47, 3 (1996), 690–5, 10.1212/wnl.47.3.690 8797466 ). Recent studies have suggested a connection between cadaver GH treatment and Alzheimer’s disease ( G. Banerjee , S.F. Farmer , H. Hyare , Z. Jaunmuktane , S. Mead , N.S. Ryan , J.M. Scott , D.J. Werring , P. Rudge & J. Collinge . ‘Iatrogenic Alzheimer’s disease in recipients of cadaveric pituitary-derived growth hormone’, Nature Medicine, 30 (2024), 394–402, 10.1038/s41591-023-02729-2 PMC1087897438287166).

45 For the UK see Tim Kelsey, ‘Doctor blocked growth hormone in 1970s: Pressure grows to compensate patients at risk of incurable brain disease’, *Independent*, 19 August 1993, https://www.independent.co.uk/news/uk/doctor-blocked-growth-hormone-in-1970s-pressure-grows-to-compensate-patients-at-risk-of-incurable-brain-disease-tim-kelsey-reports-1462154.html. On the social construction of GH as dangerous in another context see Bernat Lopez , ‘Creating fear: The social construction of human Growth Hormone as a dangerous doping drug’, International Review for the Sociology of Sport, 48, 2 (2012), 220–37,10.1177/1012690211432209 .

46

Paul Brown , Michael A. Preece , Robert G. Will , ‘”Friendly fire” in medicine: hormones, homografts, and Creutzfeldt-Jakob disease’, The Lancet, 340 (1992), 24–710.1016/0140-6736(92)92431-e1351607.

47

Ron G. Rosenfeld , Pinchas Cohen , Leslie L. Robison , Barry B. Bercu , Peter Clayton , Andrew R. Hoffman , Sally Radovick, Paul Saegner , Martin O. Savage and Jan M. Wit , ‘Long-term surveillance of growth hormone therapy’, Journal of Clinical Endocrinology & Metabolism, 97, 1 (2012), 68–72, 6822174422 10.1210/jc.2011-2294.

48 For details, see *ibid.* and Anders Tidblad , ‘The history, physiology and treatment safety of growth hormone’, Acta Paediatrica, (2021), 215–24, 10.1111/apa.15948 34028879 .

49 For an overview of the development of the GH therapy over the past 100 years, see Michael B. Ranke and Jan M. Wit , ‘Growth hormone – past, present and future’, Nature Reviews/ Endocrinology, 14 (2018), 285–300, 10.1038/nrendo.2018.22 29546874.

50

E. Stefanova , ‘Lechenie s rastezhen hormon v detska vyzrast’, Prakticheska pediatria, 16, 11 (2014), 4–6 . The list of approved indications for GH therapy grew substantially from the 1990s onwards. See Vageesh S. Ayyar , ‘History of growth hormone therapy”, Indian Journal of Endocrinology and Metabolism, 15, Supplement 3 (2011), 162–5, 163–410.4103/2230-8210.84852PMC318353022029019; Graber et al., *op. cit.* (note 40), 6–12.

51 McKelvey, *op. cit.* (note 39), 232.

52

Martin O. Savage , ‘The changing face of paediatric human growth hormone therapy’, Endocrines, 3 (2022), 419–27, 420, 10.3390/endocrines3030033 .

53

Theodore Silver , ‘The case for a post-mortem organ draft and a proposed model Organ Draft Act’, Boston University Law Review, 68, 4 (1988), 681–728, 69711650249.

54

H.E. Emson , ‘It is immoral to require consent for cadaver organ donation’, Journal of Medical Ethics, 29 (2003), 125–7, 12612796424 10.1136/jme.29.3.125PMC1733728.

55

John Harris , ‘Organ procurement: dead interests, living needs’, Journal of Medical Ethics, 29 (2003), 130–4, 13012796426 10.1136/jme.29.3.130PMC1733737.

56 Stoyan Stavru, *Veshtnopravno deistvie na smartta.* [Implications of death for property law] (Sofia: Cibi, 2008).

57

John Sutton Baglow , ‘The rights of the corpse’, Mortality: Promoting the interdisciplinary study of death and dying, 12, 3 (2007), 223–39, 224.

58
*Ibid.*, 235.

59

Thomas H. Champney , ‘The business of bodies: Ethical perspectives on for-profit body donation companies’, Clinical Anatomy, 29 (2015), 25–9, 2826474530 10.1002/ca.22643.

60 Sandor, *op. cit.* (note 3), 399.

61 Sabine Louët, ‘Dark Age medicine’, *Nature* (12 Oct. 2004), https://doi.org/10.1038/news041011-4.

62 Blizzard, *op. cit.* (note 37), 88.

63
*Ibid.*, 89.

64 For a useful comparative overview and analysis, see David E. Jefferies , ‘The body as commodity: The use of markets to cure the organ deficit’, Indiana Journal of Global Legal Studies, 5, 2 (1998), 621–58.

65 For a comparison of alternative organ procurement policies, see US President’s Council on Bioethics, Staff background paper: Organ transplantation: Ethical Dilemmas and Policy Choices (2003), https://bioethicsarchive.georgetown.edu/pcbe/background/org_transplant.html#endnote1, accessed 28 April 2025.

66
*Darzhaven vestnik* [State Gazette], no. 88, 6 November 1973 and no. 66, 23 August 1985.

67 Regulation N 1 for the extraction of cadaver organs for transplantation, *Darzhaven vestnik* no. 7, 24 January 1986.

68 Peneva, *op. cit.* (note 10), 83; Peneva et al., *op. cit.* (note 13), 99.

69
*Darzhaven vestnik*, no. 15, 22 February 1991. In 1997, article 34 was amended in line with the principle of presumed consent: *Darzhaven vestnik*, no. 12, 7 February 1997.

70 None of them could add any more details during the interviews. They barely remembered the case and they mostly spoke about its broader context: the newly acquired freedom from censorship, the opportunity to address sensitive topics and the sense of empowerment for the profession. Neither then, nor now in the interviews did they refer to the legal aspects of the case.

71 Quoted in Dorothy Nelkin and Lori Andrews, ‘Homo Economicus: Commercialization of body tissue in the age of biotechnology’, *Hastings Center Report*, September-October 1998, 30–9, 32. The court acknowledged that doctors should inform their patients in advance about possible uses of their tissues but denied Moore’s claim on part of the profit. The decision was based on the argument that giving the patient property rights would impede the progress of biotechnology (*ibid.*, 32–3; see also Stoyan Stavru, ‘Kazusat “Moore” ili zashto ne pritezhavame tyaloto si’ [The Moore case or why we do not own our bodies], *Predizvikai pravoto* (2012), https://www.challengingthelaw.com/veshtno-pravo/kazusat-mur/). Justice Stanley Mosk objected to this decision: ‘the spectre of direct abuse, of torture, of involuntary servitude haunts the laboratories and boardrooms of today’s biotechnological research industrial complex’ (quoted from Nelkin and Andrews, *op. cit.*, 33).

72

Nikolas Rose , ‘The politics of life itself’, Theory, Culture & Society, 18, 6 (2001), 1–30, 15.

73 See Pascal Grosse , ‘Crisscrossing the Iron Curtain: Clinical Trials in the German Democratic Republic, 1983-1990’, Revue d’études comparatives Est-Ouest, 49, 1 (2018), 95–125 .

74 The last decades of the twentieth century saw a major shift in the funding of biomedical research from governments to pharmaceutical, medical device and biotechnology companies: in USA for instance, the share of this funding grew from 32% in the 1980s to 65% by 2015 ( Sharon R. Kaufman , Ordinary Medicine: Extraordinary Treatments, Longer Lives, and Where to Draw the Line (Durham & London: Duke University Press, 2015), 80 ).

75 Only four of the 43 children treated with GH at the Pediatrics Institute received Kabi’s Crescormon, which was purchased rather than received in exchange for hypophyses, as was the case with the Romanian GH and Serono’s Grorm. Peneva, *op. cit.* (note 10), 193.

76 According to a 1981 report, 62 of 172 articles in the field of endocrinology (36%) were published in international journals – a recognition of their good quality. Sofia State Archive, h. 2265 (RIEGG), inv. 3, a.u. 40, sheet 1-2.

77 Information on the research activities of RIEGG in 1991. Sofia State Archive, h. 2265 (RIEGG), inv. 3, a.u. 31, sheet 52.

78

Maria Jose Guerra , ‘Bioethics at stake: The challenge of corporate science and biocapitalism’, International Journal of Feminist Approaches to Bioethics, 2, 1 (2009), 52–8, 54.

79

John Ziman , ‘”Postacademic science”: Constructing knowledge with networks and norms’, Science Studies, 9, 1 (1996), 67–80, 74.

80 See Georges Canguilhem , The Normal and the Pathological. (New York: Zone Books, 1991), 156–60 for a discussion on the association of normality with statistical frequency and social normativity in relation to Quetelet’s anthropometry proving the dependence of the ideas of normality on historically specific technical and institutional developments.

81 The biotech industry emerged in the 1970s based largely on a new recombinant DNA technique creating more than 200 new therapies and vaccines (Venkasten & Peters, *op.cit.* (note 42), 106). Its development was fostered with the Bayh-Dole Act of the US Congress in 1980, which allowed universities to retain rights in patented inventions, even if publicly funded, and to license or sell them to businesses.

82 Venkasten & Peters, *op.cit.* (note 42), 101.

83

Stefan Helmreich , ‘Species of biocapital’, Science as Culture, 17, 4 (2008), 463–78, 465.

84 Rose, *op. cit.* (note 72), 1.

85
*Ibid.*, 15.

86 Nelkin and Andrews, *op. cit.* (note 71), 31.

87

Dora Vargha , Polio across the Iron Curtain: Hungary’s Cold War with an epidemic (Cambridge: Cambridge University Press, 2018)33315356. See also Geisthövel in this issue on the international collaboration for kidney transplantations.

88 Herbert L. Heyneker, *Molecular Geneticist at UCSF and Genentech, Entrepreneur in Biotechnology*, an oral history conducted by Sally Smith Hughes, Ph.D. in 2002, Regional Oral History Office, The Bancroft Library, University of California, Berkeley (2004), 109, https://oac.cdlib.org/view?docId=hb0n39n481&brand=calisphere&doc.view=entire_text.

